# Collagen-Binding Peptidoglycans Inhibit MMP Mediated Collagen Degradation and Reduce Dermal Scarring

**DOI:** 10.1371/journal.pone.0022139

**Published:** 2011-07-11

**Authors:** Kate Stuart, John Paderi, Paul W. Snyder, Lynetta Freeman, Alyssa Panitch

**Affiliations:** 1 Weldon School of Biomedical Engineering, Purdue University, West Lafayette, Indiana, United States of America; 2 School of Veterinary Medicine, Purdue University, West Lafayette, Indiana, United States of America; University of Delhi, India

## Abstract

Scarring of the skin is a large unmet clinical problem that is of high patient concern and impact. Wound healing is complex and involves numerous pathways that are highly orchestrated, leaving the skin sealed, but with abnormal organization and composition of tissue components, namely collagen and proteoglycans, that are then remodeled over time. To improve healing and reduce or eliminate scarring, more rapid restoration of healthy tissue composition and organization offers a unique approach for development of new therapeutics. A synthetic collagen-binding peptidoglycan has been developed that inhibits matrix metalloproteinase-1 and 13 (MMP-1 and MMP-13) mediated collagen degradation. We investigated the synthetic peptidoglycan in a rat incisional model in which a single dose was delivered in a hyaluronic acid (HA) vehicle at the time of surgery prior to wound closure. The peptidoglycan treatment resulted in a significant reduction in scar tissue at 21 days as measured by histology and visual analysis. Improved collagen architecture of the treated wounds was demonstrated by increased tensile strength and transmission electron microscopy (TEM) analysis of collagen fibril diameters compared to untreated and HA controls. The peptidoglycan's mechanism of action includes masking existing collagen and inhibiting MMP-mediated collagen degradation while modulating collagen organization. The peptidoglycan can be synthesized at low cost with unique design control, and together with demonstrated preclinical efficacy in reducing scarring, warrants further investigation for dermal wound healing.

## Introduction

Scarring of the skin, a necessary component of the healing process, can have a significant psychological and aesthetic impact on a patient. Dermal scarring occurs as a result of deep dermal wounds such as those with surgical incisions, traumatic avulsions, or burn injuries, yet there are currently no products that have produced consistent results in regenerating healthy dermal tissue and preventing excessive scar tissue formation. By understanding the structural and biochemical differences between healthy tissue and the scar tissue that results from wounding, new therapeutics can be developed that reduce or prevent scarring.[1] The present work has taken such an approach by developing a synthetic collagen-binding peptidoglycan therapeutic (DS-SILY), which targets collagen fibril organization to restore elements of healthy tissue and shows promise as a new approach for inhibiting scarring in adult skin.

In nature, decorin is the most abundant proteoglycan (PG) found in adult skin.[2], [3] It is a member of the small leucine rich PGs (SLRPs) and consists of a collagen-binding protein core attached to a dermatan sulfate (DS) glycosaminoglycan (GAG) side chain. The mechanism in which decorin influences skin architecture and wound healing is multi-faceted, and includes a well-known role in binding to collagen and influencing its organization.[4]–[8] Decorin binds to collagen via its protein core, and functions to modulate collagen fibrillogenesis and the distribution of fibril diameters.[8]–[10] Multiple studies have examined the roles of decorin or its absence in skin architecture and wound healing (**Table 1**). In decorin knockout mice, collagen fibrils have irregular outlines and have a wide distribution of diameters, unlike the relatively consistent collagen fibril diameters of healthy skin.[10], [11] These knockout mice have fragile skin and exhibit impaired dermal healing.[9]–[11] Another example of impaired or abnormal healing correlating with decorin deficiency is in hypertrophic scarring; decorin is present in early stages of hypertrophic scar tissue at levels only 25% that of normal healthy tissue.[2]

**Table 1:** Comparison of effects of decorin and DS-SILY peptidoglycan in wound healing.

Mechanism of Action	Decorin	Peptidoglycan
Incorporate bioactive GAG chain	[15]–[17]	[15]–[17]
Control collagen fibrillogenesis	[4], [6], [8], [10], [39], [43]	[5]
Enhance mechanical strength	[5], [6], [8], [10]	[5]
Prevent MMP-mediated collagen degradation	[18]	Present work
Inhibit TGFβ activity	[22], [25]–[27], [44]	

Collagen organization within tissues and in tissue engineered matrices is a critical parameter that affects cellular behavior.[12] In fact modulating collagen organization using collagen-binding peptides alone may improve healing by regulating collagen fibril diameter and thus enhancing the tensile strength of the skin.[13], [14] Incorporating the DS GAG chain of decorin may further improve healing through its host of biochemical activities.[15] For example, DS binds and promotes function of fibroblast growth factors 2 and 10 (FGF-2, FGF-10), which promotes the proliferation of cells that function in response to injury.[16], [17] In addition to its role in biochemical signaling, the negatively charged DS also provides a hydrated wound environment which improves healing by reducing water loss and restoring homeostasis to the scar.[14]

Decorin also plays a critical role in the balance of inflammation, collagen synthesis, and collagen degradation, which are critical parameters in the healing process. Recent work has shown that decorin can protect collagen fibrils from collagenase degradation.[18] Collagenases are members of the matrix metalloproteinase (MMP) family, which are upregulated during wound healing and can initiate the degradation of mature fibrillar type I collagen, which may increase scarring.[19], [20] Additionally, decorin has been shown to bind to and lower transforming growth factor beta (TGFβ) levels in wounded tissue, attenuating the inflammatory response and decreasing scar formation.[21]–[27]

There is strong evidence that decorin plays a critical role in tissue healing, however decorin is difficult to isolate from animal tissues and to manufacture, so clinical investigation of decorin in wound healing is limited.[27], [28] We have therefore developed a synthetic collagen-binding peptidoglycan, modeled after decorin, which contains a collagen-binding peptide attached to the biochemically-active DS GAG chain. Unlike decorin, the synthetic peptidoglycan is highly tailorable and can be manufactured with relative ease.[5] Like decorin, the synthetic peptidoglycan has been shown to modulate collagen architecture in vitro, and through its binding to collagen, can act as physical barrier to platelet adhesion and subsequent activation.[5], [29] Here we further investigate the function of the synthetic peptidoglycan by demonstrating its ability to inhibit MMP mediated collagen degradation and mitigate dermal scarring in a rodent model of dermal wound healing.

## Materials and Methods

### Reagents

Peptide RRANAALKAGELYKSILYGC (SILY) was purchased from Genscript (Piscataway, NJ). Dermatan sulfate (DS) and periodate oxidized DS (oxDS) (41kDa and 6.85% sulfur) were purchased from Celsus Laboratories (Cincinnati, OH). Crosslinker PDPH (3-[2-Pyridyldithio]propionyl hydrazide) was purchased from Pierce (Rockford, IL). Hyaluronic acid (HA) 5 mg/mL, MW>1 *10^6^ Da (Hycoat) was purchased from Neogen (Lexington, KY). Rat tail collagen was purchased from BD Biosciences (Bedford, MA). MMPs were purchased from R&D Systems (Minneapolis, MN). All other supplies were purchased from VWR (West Chester, PA) or Sigma-Aldrich (St. Louis, MO) unless otherwise noted.

### Peptidoglycan synthesis

Peptidoglycan DS-SILY was synthesized as previously described.[5] Briefly, oxDS was coupled to the heterobifunctional crosslinker PDPH forming DS-PDPH. Excess PDPH was removed by size-exclusion chromatography and DS-PDPH was reacted with peptide SILY yielding the collagen-binding synthetic peptidoglycan DS-SILY. The peptidoglycan was separated from excess free peptide by size exclusion chromatography using MilliQ running buffer. DS-SILY was lyophilized and stored at −20°C until further testing.

### In vitro collagen degradation

Collagen degradation studies were performed as previously described by Geng and colleagues, and according to the manufacturer's instructions for the MMPs.[18] Collagen was diluted to 0.4 mg/mL in 10 mM HCl and was diluted in equal volumes with TES buffer (N-[tris(hydroxymethyl)methyl]-2-aminoethanesulfonic acid, 2-[(2-hydroxy-1,1 bis(hydroxymethyl)ethyl)amino]ethanesulfonic acid) and vortexed thoroughly to mix. Fibrils were precipitated by incubating 1 hr at 37°C, and were then pelleted by centrifuging at 12,000 rpm for 1 minute. The supernatant was removed and the pellet resuspended in an equal volume of TES buffer. Vortexing and sonication were required to break up the fibril pellet for a homogenous mixture of collagen. The homogenous collagen solution was split into 25 µL aliquots for treatment.

To untreated samples, 25 µL water was added to the collagen solution (NT). To peptidoglycan treated samples, 15 µL water and 10 µL synthetic peptidoglycan (1.83 mg/mL) was added to the collagen solution (DS-SILY). The collagen solutions were incubated for 1 hour at room temperature to allow binding. Samples were then pelleted by centrifugation at 15,000 rpm for 5 minutes and the supernatant was removed.

The degradation buffer was made within 1 hour of collagen degradation studies. To the tris buffer, APMA (amino-phenyl mercuric acetate) (3.5 mg/mL in 0.1 M NaOH) was added to a final concentration of 1 mM APMA. HCl (1 M) was added at one tenth the amount of APMA solution to maintain pH at 7.6. Collagen pellets were resuspended in 25 µL water and 25 µL degradation buffer. To control samples, 1 µL 1X PBS was added, and to the degradation samples, 1 µL MMP-1 or MMP-13 (0.2 mg/mL and 0.5 mg/mL, respectively) was added. Samples were mixed by vortexing and were incubated at 32°C overnight, then frozen until further testing.

Samples were run under reducing conditions on a 7.5% Precast Polyacrylamide gel (Biorad Tris 7.5% Mini-PROTEAN TGX Precast Gel) at 200V. Protein in the gel was stained with Coomassie Orange for 45 minutes, rinsed twice with 7.5% acetic acid, rinsed 5 minutes with water, and imaged using a trans-UV source. Protein bands were analyzed using ImageJ software and the percent degradation was determined by comparing the intensity of the total intact bands including alpha 1, alpha 2, and oligomer bands. Collagen bands in samples treated with MMP-1 or with MMP-13, with or without prior peptidoglycan incubation, were compared to those same bands in collagen controls not treated with MMP.

### Rat incisional model

For the animal study, HA was used as a viscous delivery vehicle for the peptidoglycan. Peptidoglycan DS-SILY was dissolved in the HA vehicle at a concentration of 0.5 mg/mL, a dose approximately equal to 10 µM in which maximal binding to collagen is achieved.[29]

Male Long-Evans rats were purchased from Harlan Labs (San Diego, CA) and were handled according to approved Purdue Animal Care and Use Committee (PACUC) protocol 08-111. Rats were housed in individual cages and acclimated for 1 week prior to surgery. At time of surgery, rats were anesthetized by chamber delivery of isoflurane and oxygen and their backs shaved and aseptically prepared. A single 4 cm longitudinal incision was made through the skin and panniculus muscle along the dorsal spine. A single dose of 250 µL treatment (no treatment (NT), HA, or DS-SILY) was applied to the incision and the wound was closed by simple interrupted absorbable sutures. Each treatment group contained 3 rats for each time point.

Following surgery the rats recovered in their individual cages and were given two injections of buprenorphine for pain. The rats were observed daily and one rat was replaced due to complications from self mutilation 24 hours after surgery.

At 3, 7, 10, 14, and 21 days, three rats in each group were euthanized and a necropsy was performed. The back was shaved and the incision was photographed. The skin containing the healing incision was then excised and cut into eight 4 mm strips perpendicular to the incision using a custom cutting device with fixed blades. Alternate skin samples were immediately fixed in 10% neutral buffered formalin for histological analysis or were placed in cold 1xPBS and immediately tested for tensile strength.

### Tensile strength testing

The skin samples were loaded into the grips of a mechanical testing system (Test Resources, model:100P/Q) such that the incision line was orthogonal to the direction of tension. The grips were set at a distance of 0.5 in and the scar was centered between the grips. Samples were then loaded under tension at a rate of 5 mm/min to failure. The ultimate tensile strength at failure (breaking strength) was recorded.

### Histological evaluation

Formalin preserved skin samples were routinely processed and sectioned for H&E and Masson's Trichrome staining. H&E stained samples were examined for inflammation by a board certified pathologist using a scale adapted from Simhon and coworkers, which includes evaluation of re-epithelization in the form of hyperplasia, the degree and type of inflammatory cells present, the appearance of fibroblasts and foreign body giant cells, and the presence of granulation tissue.[30] Trichrome stained images were scanned and the wound area was quantified using ImageJ software. The granulation tissue was defined by characteristic immature collagen with parallel orientation and dense packing, described by Beausang and coworkers.[31] The total area composed of this granulation tissue was traced and quantified

### Transmission electron microscopy (TEM)

Histological samples were further processed for TEM imaging. Slides of sections stained with Masson's Trichrome were used to identify and trim the paraffin tissue block to contain only the area of the healing incision. Samples were deparaffinized by incubating in toluene for 3 hours. Samples were embedded and cut 0.5 mm deep, horizontal to the epidermal surface. Cross sections of collagen fibrils were imaged using an FEI/Philips CM-10 transmission electron microscope (FEI Company, Hillsboro, OR) using an accelerating voltage of 80 kV and at a magnification of 52 K. Three images were taken of each wound from each rat. The diameter of each fibril in each image was measured using ImageJ software.

### Visible scar assessment

To evaluate visible scarring at 21 days, rats (n = 9 rats per group) were shaved and incisions were photographed. Photographs were taken using a camera stand with a fixed distance, external lighting, and included ruler for scale. The digital images were loaded into ImageJ software and five observers were trained to trace visually scarred areas using the line tool. If the visible scar was not continuous, the length of each scar segment was added together to find a total visible scar length. Overall trends between treatment types was the same for each observer, however the raw values obtained for measured visible scar varied by observer. In order to account for these differences, lengths were normalized to untreated wounds for each observer and percent increase/decrease over untreated wounds was compared. In this way, measurements from each observer could be combined for analysis. Inter-observer variability was small when comparing these normalized values for scar length.

### Statistics

All observers were blinded to treatment type. The breaking strength, wound area, and visible scar length were compared on 3 levels: no treatment, HA control, and DS-SILY. The TEM analysis was performed on these 3 levels and compared to intact skin. Quantitative data was normally distributed and statistics were analyzed by ANOVA using Design Expert software (StatEase, Minneapolis, MN). Results are presented as average + S.D. and significance was set by α = 0.05.

## Results

### In Vitro collagen degradation

**Figure 1:**
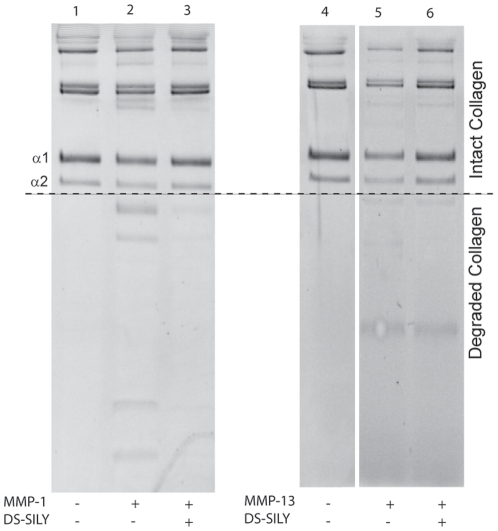
Collagen degradation by MMP. Intact collagen bands, shown above the dotted line, were quantified and used to determine the amount of collagen degraded, while collagen degradation products appear below the dotted line. Collagen degradation with MMP-1 was decreased with pre-incubation of DS-SILY from 18% to 1% (columns 2 and 3). Collagen degradation with MMP-13 was decreased with pre-incubation of DS-SILY from 55% to 15% (columns 5 and 6). Intact collagen not incubated with MMP-1 or MMP-13 (columns 1 and 4) was used for comparison.

Peptidoglycan was added to collagen fibrils and then degradation by MMPs was assessed. As shown in **Figure 1**, the alpha 1 and alpha 2 chains of type I collagen and the heavier oligomer bands are considered to be intact collagen and are indicated above the dotted line. Degraded collagen, as indicated by smaller molecular weight bands, appears below the line. Columns 1 and 4 depict collagen that was not subjected to degradation with either MMP. The intensity of the intact bands (alpha 1, alpha 2, and oligomer bands) in these lanes was used as a baseline. MMP treatment (columns 2 and 5) degrades intact collagen and results in the presence of smaller molecular weight degradation bands, and correspondingly less intense intact bands. The intensities of the intact bands were measured and revealed that MMP-1 degraded 18% of the intact bands, while MMP-13 degraded 55% of the intact bands. Incubating the collagen with DS-SILY prior to MMP treatment decreased degradation of the intact collagen bands to 1% with MMP-1 and 15% with MMP-13 (columns 3 and 6). A separate study was performed in which DS-SILY was added to untreated collagen at the same time as MMP-1 or MMP-13 to verify that DS-SILY itself did not inhibit MMP activity (data not shown).

### Tensile strength

**Figure 2:**
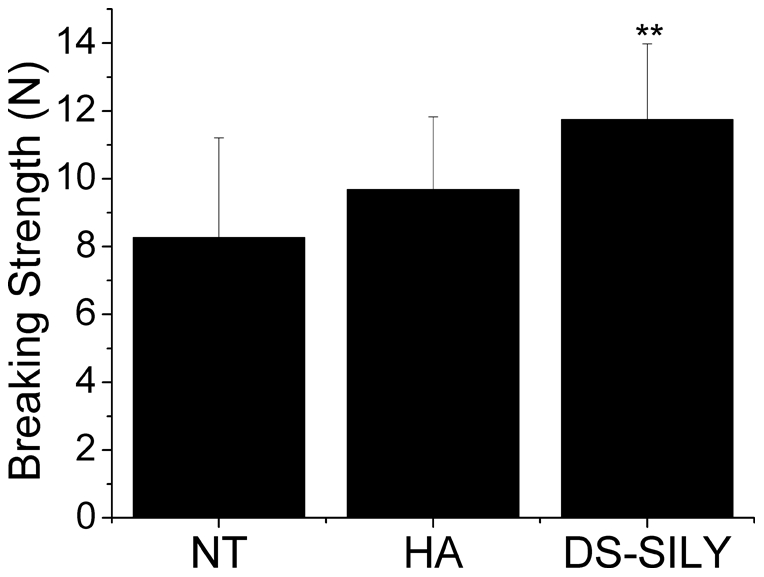
Tensile Strength. Mean breaking strength of healed incisions from each group of rats 21 days after surgery. ** denotes that DS-SILY treated wounds were significantly stronger than both non treated controls (NT) (p<0.001) and those receiving the HA excipient (HA) (p = 0.0178). n = 12 tissue samples (3 rats in each group).

All of the rats recovered from surgery and survived to the time of necropsy. There was no evidence of incisional infection or self-mutilation. From 3 to 21 days, all wounds became progressively stronger; however, there were no significant differences between the treatment groups at 3, 7, 10, or 14 days (data not shown). At 21 days, the DS-SILY treated wounds were significantly stronger than untreated (NT) or HA treated wounds (**Figure 2**). HA treatment showed a modest, but not significant, increase in wound strength over untreated wounds.

### Histologic evaluation

**Figure 3:**
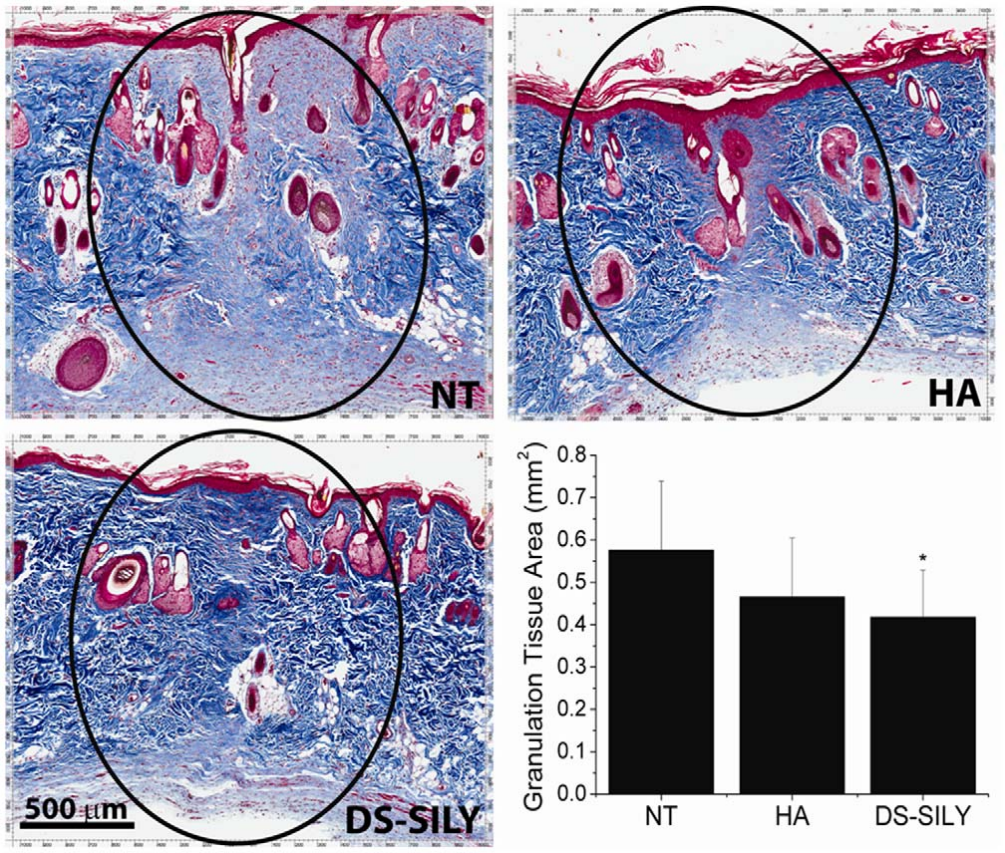
Histology. Representative Mason Trichrome stained microscopic sections of healed incisions in rats at 21 days. Arrows indicate the outer limit of the granulation tissue area with no treatment (NT), HA excipient (HA), and peptidoglycan-treated wounds (DS-SILY). In each group, note the marked differences in collagen organization and maturity in the granulation tissue area. The area of granulation tissue in each image was measured in ImageJ. * denotes that DS-SILY treatment resulted in significantly smaller wound areas as compared to untreated wounds (NT) (p = 0.0109). n = 11 tissue samples.

There were no notable differences in the inflammatory response between peptidoglycan-treated wounds and control wounds (data not shown), indicating that the peptidoglycan did not cause any adverse tissue reaction in the healing incision. Representative images of trichrome stained incisions from each treatment group are shown in **Figure 3**. The 21-day samples were trichrome stained and the total area of granulation tissue was measured, showing that incisions receiving peptidoglycan treatment had significantly less granulation tissue than untreated wounds (**Figure 3**). These results were confirmed using a qualitative scoring method that has been used to evaluate collagen organization, maturity, and density; [31] the scores followed the same trends as for total granulation tissue area (data not shown).

### Collagen fibril diameter

**Figure 4:**
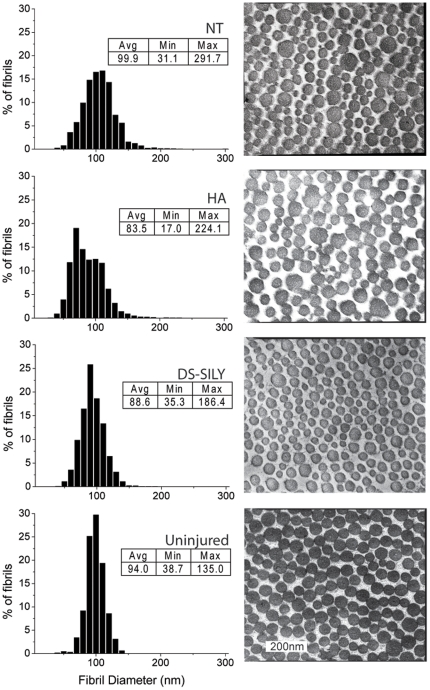
Collagen Fibril Diameter by TEM. Representative TEM images of collagen fibrils from wounded areas and histograms of fibril diameters. There was a broader distribution of collagen fibril diameters in untreated and HA treated incisions. The DS-SILY treated wounds more closely resembled intact skin with a narrower distribution of collagen fibril diameters. Graph inserts indicate treatment type, average fibril diameter (nm), and the total range of fibril diameters (nm).

Sections of uninjured skin and the healing incisions at 21 days post injury were processed for TEM and assessed for collagen fibril diameter. **Figure 4** shows representative images of cross sections of collagen fibrils and histograms of the collagen fibril diameters from each group of rats and from intact skin. Each graph inset indicates the treatment type, average fibril diameter, and range of fibril diameters (nm). Untreated wounds and HA control wounds contained collagen fibrils with a wide range of diameters. Treatment with DS-SILY resulted in collagen fibril diameter and distribution similar in appearance to intact skin.

### Visible scar

**Figure 5:**
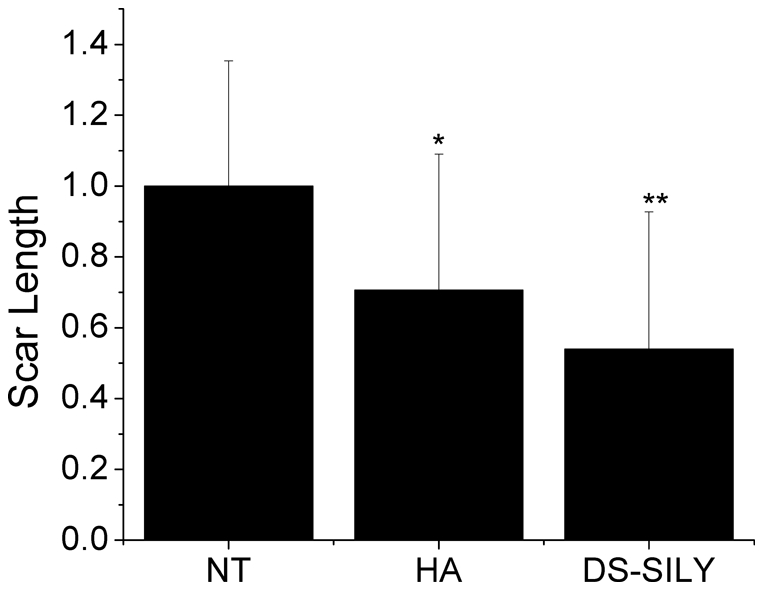
Visible Scar Length. Graph comparing the visible scar lengths remaining 21 days after surgery in 3 groups of rats. The average scar length remaining from wounds receiving no treatment (NT) was set to a length of 1.0 and the visible lengths of the other wounds were expressed as fractions of the average NT length. * denotes that wounds receiving no treatment had significantly high visible scars as compared to wounds receiving HA (p = 0.0005). ** denotes that wounds receiving DS-SILY decreased the visible scar compared to both NT (p<0.0001) and to HA (p = 0.0372). n = 45 measurements.

**Figure 5** shows the visual scar length remaining 21 days after surgery. The HA treated wounds showed significantly less visible scar compared to untreated wounds. The peptidoglycan (DS-SILY) treated wounds showed significantly less visible scar length compared to both the untreated wounds and wounds receiving the HA control.

## Discussion

The native proteoglycan, decorin, plays a critical role in tissue healing and in collagen organization.[6], [9]–[11] Because decorin is difficult to manufacture and its use limited, we have designed a synthetic collagen-binding peptidoglycan which mimics, in part, the functions of decorin (**Table 1**). The aim of this study was to investigate the ability of the synthetic peptidoglycan DS-SILY to inhibit MMP-mediated collagen degradation, and to assess the resulting effects on dermal healing in the rat model.

Previously, our group reported the ability of DS-SILY to modulate collagen fibril organization and increase the stiffness of collagen gels in a manner similar to decorin.[5] Recently, Geng and coworkers showed that native proteoglycans, including decorin, fibromodulin, and lumican, protect collagen from MMP degradation. Additionally, other PGs such as aggrecan play a protective role against enzymatic degradation of the ECM. We hypothesized that the synthetic peptidoglycan would have a similar effect to native PGs in protecting collagen from degradation against collagenases.[18] There are numerous MMPs involved in the wound healing process. MMP-1 and MMP-13, which are upregulated following injury, cleave intact collagen.[19], [32]–[34] The degraded collagen is then replaced with new collagen, increasing granulation tissue volume and thus giving rise to a more visible scar. Like native decorin, the synthetic peptidoglycan prevents MMP-1 and MMP-13 mediated collagen degradation by binding to collagen fibrils and providing a protective mask.[18], [29] Other MMPs, including MMP-8, are upregulated both in normal healing and non-healing wounds, and the ability of a peptidoglycan to protect collagen from degradation by these other MMPs is currently under investigation.[19], [20], [35] Additionally, the concentration of MMPs used in this study did not fully degrade the collagen. The ability of the peptidoglycan to protect collagen from higher concentrations of the MMPs will also be examined in future studies.

Because the synthetic peptidoglycan DS-SILY inhibited MMP collagen degradation in vitro, coupled with its demonstrated ability to modulate collagen organization, we further investigated its application in healing of dermal wounds. Incisional wounds were created in rats, and the effects of the peptidoglycan on wound healing were studied over 21 days. HA was used as a viscous delivery vehicle so that the peptidoglycan would remain in place allowing time for the peptidoglycan to bind to exposed collagen while the wound was sutured. HA has been suggested to enhance dermal healing by providing a hydrating wound environment and through its host of biochemical and biomechanical functions, and as such, was chosen over other relatively inert excipients such as pluronic.[36], [37] Our study included both HA and untreated control groups for comparison.

Collagen organization in the incisional scar was evaluated histologically and by TEM. By both measures, peptidoglycan treated wounds resulted in improved collagen morphology, with uninjured skin tissue used as a standard. Histological analysis revealed that the total area of granulation tissue in wounds treated with peptidoglycan was significantly reduced compared to wounds that were not treated. These results support the hypothesis that by inhibiting MMP mediated collagen degradation at the injury site, the synthetic peptidoglycan DS-SILY decreases the total amount of granulation tissue.

TEM analysis revealed that while the HA treated wounds contained collagen fibrils with the smallest average diameter, the distribution of diameters in these wounds was spread with some collagen fibrils over 200nm. Only wounds that were not treated contained such large collagen fibrils, and a similar wide distribution of fibril diameters. In contrast, the peptidoglycan treated wounds did not contain any of these abnormally large fibrils and had a distribution of fibril diameters more closely resembling that of uninjured skin. These results corroborate our previous in vitro studies and indicate that the synthetic peptidoglycan regulates collagen organization and prevents lateral aggregation of collagen fibrils in a manner similar to decorin.[5], [12]

Scar tissue is weaker than healthy uninjured skin, which is a consequence of immature collagen with nonuniform architecture and organization.[38] Therefore the breaking strength of the scar is an objective and quantifiable measure of tissue organization. Peptidoglycan treated wounds had increased breaking strength compared to untreated and HA control wounds, supporting the histological and TEM data, and demonstrating a functional consequence of improved collagen organization and decreased scar tissue in peptidoglycan treated wounds.

Visible scarring on the skin surface is of high patient concern. Our results demonstrated an improved cosmetic outcome with peptidoglycan treatment and positively correlated with decreased granulation volume seen histologically. Visible scarring in rats is apparent by the lack of hair and a pale and somewhat shiny epidermis. Since the peptidoglycan functions by regulating collagen architecture and by inhibiting MMP mediated degradation of intact collagen, it is likely that decreased visible scarring is a result of decreased granulation volume rather than a restoration of all of the components of healthy skin including hair follicles. While animal models are somewhat limited for assessing dermal scarring, decreasing granulation tissue volume in human skin is likely to have a similar positive impact on cosmetic outcome.

For this study, HA was used as a viscous excipient for delivery of DS-SILY. HA treated control wounds showed trends towards increased breaking strength, decreased wound area, and improved histological evaluation over the untreated incisions. There was a significant improvement in visible scarring and these results may indicate that HA alone offers some benefits in enhancing dermal healing. Some investigations suggest that the water inclusion provided by HA can facilitate the migration of cells to the wound area and facilitate movement of newly synthesized collagen molecules and fibrils.[39] Our study shows that while HA offered some benefits to the healing wound, it does not affect the uncontrolled lateral aggregation of collagen fibrils that leads to nonuniform architecture and organization. The increased hydration with HA, combined with the ability of DS-SILY to both protect collagen from degradation and control collagen architecture, may have acted synergistically to prevent dermal scarring. Further studies are needed to determine the ideal excipient for DS-SILY and to isolate the unique mechanisms of action of the peptidoglycan.

Other work in dermal healing has highlighted the inflammatory aspect of wound healing and its contribution to scar formation. In particular, studies have focused on the impact of reducing levels of TGFβ1 during the inflammatory phase of wound healing in order to reduce scar formation.[21], [23]–[25], [27], [40]–[42] The core protein of decorin is able to bind to and inhibit TGFβ1, and could be a possible treatment for scar reduction by this mechanism. The synthetic peptidoglycan, DS-SILY, while modeled to mimic many of the functions of decorin, does not contain the large core protein of decorin, and apparently cannot operate by inhibiting TGFβ1 in the wound bed. The peptidoglycan does mimic many of the other functions of decorin that may play a role in dermal healing, and offers advantages over native decorin in its ease and low cost of manufacturing.

Recognizing the limitations of using normal animal models to study wound healing, the results of this study warrant further investigation. Here a single dose of peptidoglycan applied at the time of injury and immediately prior to suturing resulted in improved dermal healing as defined by greater tensile strength, improved collagen organization, and improved visible scar 21 days following a linear incision in the rat. This peptidoglycan may offer a feasible approach for scar mitigation and further investigation for human use is warranted.
